# Desmoplastic Small Round Cell Tumor with “Pure” Spindle Cell Morphology and Novel *EWS-WT1* Fusion Transcript: Expanding the Morphological and Molecular Spectrum of This Rare Entity

**DOI:** 10.3390/diagnostics11030545

**Published:** 2021-03-18

**Authors:** Gaetano Magro, Giuseppe Broggi, Angelica Zin, Vincenzo Di Benedetto, Mariaclaudia Meli, Andrea Di Cataldo, Rita Alaggio, Lucia Salvatorelli

**Affiliations:** 1 Department of Medical and Surgical Sciences and Advanced Technologies, “G. F. Ingrassia”, Anatomic Pathology, University of Catania, 95123 Catania, Italy; giuseppe.broggi@phd.unict.it (G.B.); lucia.salvatorelli@unict.it (L.S.); 2 Institute of Pediatric Research “Città della Speranza”, 35127 Padova, Italy; a.zin@irpcds.org; 3 Department of Medical and Surgical Sciences and Advanced Technologies, “G. F. Ingrassia”, Pediatric Surgery Unit, University of Catania, 95123 Catania, Italy; vdb@chirpedunict.it; 4 Pediatric Oncohematology Unit, Department of Clinical and Experimental Medicine, University of Catania, 95123 Catania, Italy; mclaudiameli@gmail.com (M.M.); adicata@unict.it (A.D.C.); 5 Pathology Unit, Bambino Gesù Children’s Hospital, IRCCS, 00165 Rome, Italy; ral@unipd.it

**Keywords:** desmoplastic small round cell tumor, pediatric tumors, soft tissue tumors, diagnosis, EWS-WT1 fusion gene

## Abstract

Background: Desmoplastic small round cell tumor (DSRCT) is a rare pediatric soft tissue neoplasm composed of small round tumor cells with prominent stromal desmoplasia, polyphenotypic differentiation and *EWSR1-WT1* gene fusion. We, herein, present a unique case of DSRCT, exhibiting a pure spindle cell morphology, absence of desmoplastic stroma and showing a novel *EWS-WT1* fusion transcript. Methods: A 12-year-old boy presented multiple intra-abdominal, confluent and mass-forming nodules that affected the entire abdominal and pelvic cavities. Results: Histologically, the nodules were composed of spindle cells with scant cytoplasm and oval nuclei arranged into short, intersecting fascicles and set in a scant, non-desmoplastic, stroma. Immunohistochemically, neoplastic cells were stained with vimentin, desmin, WT-1 (C-terminus antibodies) and EMA. Reverse-transcriptase polymerase chain reaction (RT-PCR) analysis showed the presence of an unusual chimeric transcript, composed of an in-frame junction of exon 9 of *EWS* to exon 7 of *WT1*, confirming the histological diagnosis of DSRCT. Conclusions: The present case contributes to widen the morphological spectrum of this entity; notably, the additional presence of a novel chimeric fusion transcript contributes to making the present case even more unique. Whether the detection of the above-mentioned fusion transcripts could explain the unusual morphology of the tumor remains to be established.

## 1. Introduction

Desmoplastic small round cell tumor (DSRCT) is an extremely rare and aggressive round cell sarcoma, first described by Gerald and Rosai in 1989 [1] and genetically defined by a peculiar molecular alteration involving chromosomes 11p13 and 22q12 resulting in *EWSR1-WT1* fusion gene that encodes an aberrant transcription regulatory factor consisting of the trans-activation domain (N-terminal portion) of EWS and the DNA binding domain (C-terminal portion) of WT1 [2,3,4,5,6]. DSRCT mostly affects children and young adult males and it clinically presents as abdominal/pelvic multinodular masses with diffuse peritoneal involvement, mimicking peritoneal carcinomatosis [7,8,9]. Other unusual sites can be occasionally involved, including thorax, lymph nodes, pleura, lung, kidney, parotid gland and orbit [10,11,12,13]. Although the treatment is extremely aggressive (surgery, chemotherapy, radiotherapy), survival rate remains poor (three-year rate and five-year rate of 44%, and 15%, respectively) [14].

Histologically, DSRCT consists of irregular, small nests of round, undifferentiated neoplastic cells with scant cytoplasm and hyperchromatic nuclei, set in an abundant desmoplastic stroma [15]. It characteristically exhibits a polyphenotypic immunohistochemical profile, with co-expression of epithelial and mesenchymal markers, including cytokeratins, EMA, vimentin and desmin [15,16,17,18,19]. In addition, DSRCT shows a peculiar nuclear expression of C-terminus portion of WT1 protein, that currently represents the most specific immunomarker [15,16,17,18,19,20]. The diagnosis of DSRCT is relatively straightforward if the tumor arises in typical sites, exhibiting both its characteristic morphological and immunohistochemical features; conversely, diagnostic difficulties may arise when tumor occurs at unexpected sites and/or if it shows unusual morphology and immunohistochemical profile. The most diagnostically challenging features are related to a spindle cell morphology, ranging from focal to diffuse, or to the absence of desmoplastic stroma [21,22,23]. In most cases the *EWS-WT1* fusion transcript consists of the first 7 exons of the *EWS* gene and the last three exons (exons 8–10) of the *WT1* gene (*EWS-WT1* 7/8) [24]. However, several alternative breakpoints for the *t(11;22)(p13;q12)* translocation have been described [25,26,27,28,29]. The resulting fusion transcripts generally contain additional exons from *EWS* with conservation of the *WT1* complement (*EWS-WT1* 8/8, 9/8, and 10/8) [28].

We, herein, report the first case of intra-abdominal DSRCT characterized by a “pure” spindle cell morphology with no desmoplastic stromal reaction, associated with a novel chimeric transcript, composed of an in-frame junction of exon 9 of *EWS* to exon 7 of *WT1*. If the unusual morphology of this tumor is related to its different fusion transcript remains to be established.

## 2. Materials and Methods

### 2.1. Clinical Features

**Figure 1:**
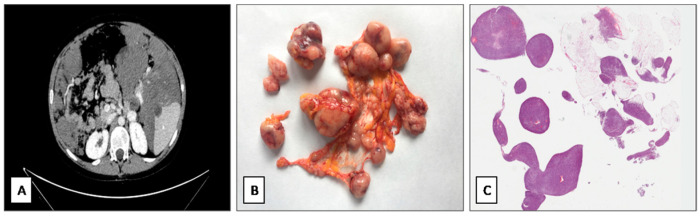
(**A**) Computer Tomography imaging showing multiple intra-abdominal, confluent and mass-forming nodules, affecting the entire abdomen and pelvis. (**B**) Gross examination showing multiple, whitish in colour, omental nodules. (**C**) Histological examination. Low magnification showing the histologic correspective of the gross appearance of the tumor: multiple hypercellular nodules within omental tissue (hematoxylin and eosin; original magnification 25×).

A 12-year-old boy had been followed at our centre for a history of epistaxis and gingival bleeding. His family history was positive for parotid and breast carcinoma (grandmother, on mother side) and Ewing sarcoma (cousin on the mother side). At the hospital admission, he presented persistent thrombocytopenia and leukopenia (PLT 25.000 mmc, WC 2590 mmc) for six months. Bone marrow aspirate presented low cellularity and dysmielopoiesis notes. It was also performed abdominal and neck ultrasound that showedcervical region pathological lymphadenopathy. Lymphocyte subpopulation analysis revealed 3.4% of CD3+TCR alphabeta+CD4−CD8−. T-lymphocytes survey after FAS stimulation was 15%. A clinical diagnosis of *“autoimmune lympho-proliferative syndrome”* (ALPS) [30] was rendered and the patient was treated at first with prednisone (15 mg/daily) and then, for the persistence of the symptoms, also with mycophenolate mofetil (800 mg/daily) that led to a partial and temporary therapeutic response. After a 40 months-follow-up, he presented constipation, globose abdomen and vague abdominal pain. Radiological examination (ultrasound and Computed Tomography) revealed an abdominal mass in right hypochondrium, as well as multiple intra-abdominal, confluent and mass-forming nodules that affected the entire abdominal and pelvic cavities (Figure 1A). Intra-abdominal lymphoma was suspected. Patient underwent laparotomy with multiple omental biopsies. Tissue samples were formalin-fixed, paraffin-embedded and stained with hematoxylin & eosin (H&E). Immunohistochemical analyses were performed using the standard avidin-biotin-peroxidase method using the Dako automated immunostainer (Dako autostainer link 48, Glostrup, Denmark). A wide panel of antibodies was tested, including vimentin, desmin, myogenin, MyoD1, WT-1 (C-terminus), WT-1 (N-terminus), INI-1, NB84, alpha-smooth muscle actin, pan-cytokeratins, EMA, CD117, CD99, CD56, LCA, CD30, S-100 protein, CD34, STAT-6, Chromogranin A and Synaptophysin.

### 2.2. RT-PCR

Reverse-transcriptase polymerase chain reaction (RT-PCR) assays were performed to detect *EWS-WT1* fusion transcript, as previously reported [31]. The quality of RNA and efficiency of reverse transcription were assessed by analyzing the expression of *beta2-microglobulin*. 

## 3. Results

### 3.1. Histological and Molecular Findings

**Figure 2:**
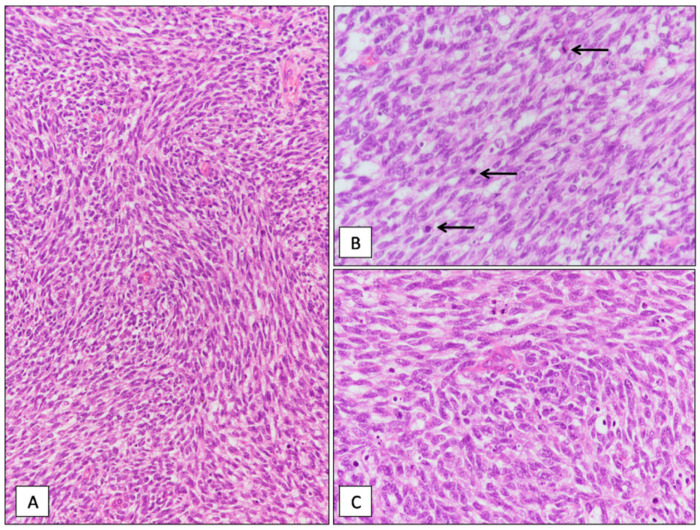
Histological examination. (**A**) The tumor was composed of closely packed spindle cells with scant cytoplasm and oval nuclei, arranged into short intersecting fascicles with scant interposed stroma (hematoxylin and eosin; original magnification 150×). (**B**) Higher magnification showing moderate nuclear atypia and three mitoses (arrows) (hematoxylin and eosin; original magnification 400×). (**C**) Numerous neoplastic apoptotic cells are seen (hematoxylin and eosin; original magnification 400×).

**Figure 3:**
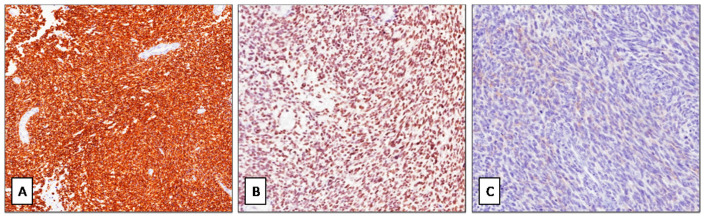
(**A**) Neoplastic cells exhibiting a strong and diffuse staining for desmin (immunoperoxidase; original magnification 100×). (**B**) Diffuse nuclear staining is obtained with WT-1 (**C**)-terminus antibody) (immunoperoxidase; original magnification 150×). (**C**) Neoplastic cells are focally and weakly stained with EMA; (immunoperoxidase; original magnification 150×).

**Figure 4:**
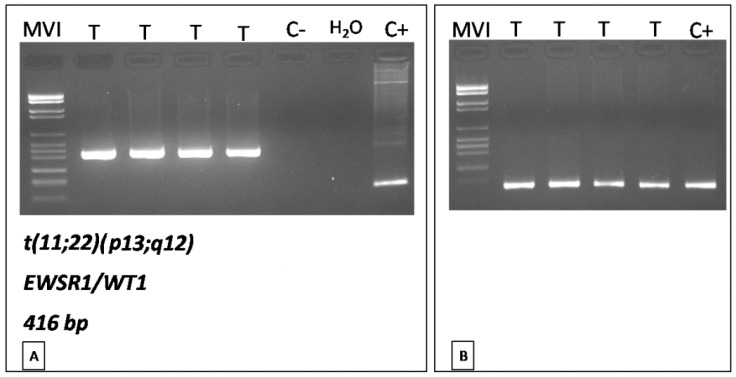
(**A**) RT-PCR analysis revealing the presence of the EWSR1-WT1 chimeric transcript, characteristic of desmoplastic small round cell tumor (MVI, marker VI; T, tumor, C-, negative control; C+, positive control). (**B**) Quality of RNA and efficiency of reverse transcription were assessed by analyzing the expression of beta2-microglobulin.

Grossly, multiple omental nodules (0.2 to 4 cm in greatest diameter), whitish in color and firm in consistency, were seen (Figure 1B). Histologically, the nodules were composed of closely packed, small- to medium-sized spindle cells with scant cytoplasm and oval nuclei with finely dispersed chromatin and inconspicuous or no nucleoli (Figure 1C). Neoplastic cells were arranged into short intersecting fascicles with a fascicular (Figure 2A) or whorling growth pattern and set in a scant, focally myxoid, stroma. Multiple foci of tumor necrosis, 28 mitoses per 10 high-power fields (HPFs) (Figure 2B) and numerous apoptotic neoplastic cells (Figure 2C) were seen. No atypical mitoses were found. Only focally a mild to moderate nuclear atypia was seen. Desmoplastic stroma was lacking. Tumor nodules showed an infiltrative growth pattern with extension into the omental adipose tissue. Due to the cytological and architectural features, the overall morphological appearance was closely reminiscent of adult-type fibrosarcoma. Immunohistochemically, neoplastic cells exhibited a diffuse expression of vimentin, desmin (Figure 3A), WT-1 (nuclear staining with C-terminus antibodies) (Figure 3B), INI-1 and focal staining for EMA (Figure 3C). The remaining antibodies tested were negative. Based on the clinical presentation (age of patient; multiple omental nodules) and the characteristic polyphenotypic immunoprofile (co-expression of vimentin, desmin, WT-1 and EMA) the diagnosis of “DSRCT with unusual morphology” was rendered, suggesting molecular analyses for further confirmation. RT-PCR analysis, using the primers for EWS (exon 7—NCBI Reference Sequence NM_005243.3) and WT1/9 (exon 8—NCBI Reference Sequence NM_000378.5), revealed a 416-bp product in the patient tissue specimen (Figure 4). DNA sequence analysis of the PCR product confirmed that the chimeric transcripts were composed of an in-frame junction of exon 9 of EWS to exon 7 of WT1. This molecular finding supported the histological diagnosis. Differential diagnosis mainly included adult-type fibrosarcoma, leiomyosarcoma and monophasic synovial sarcoma. However, unlike DSRCT, these malignant tumors usually do not arise primarily in the abdomen of adolescents as multiple nodular masses, fail to express nuclear WT1 (C-terminus antibodies) and do not show EWSR1-WT1 fusion by RT-PCR.

### 3.2. Outcome

The patient was treated according to the protocol EpSSG 2005. After six months, radiological examination revealed a slight reduction of the tumor masses. However, seven months after the diagnosis, because of the evidence of disease progression, a second-line therapy with vinorelbine and cyclophosphamide was administered with no significant results. He died 9 months after the diagnosis.

## 4. Discussion

Although the current World Health Organization (WHO) defines DSRCT as a “malignant mesenchymal neoplasm composed of small round tumor cells with prominent stromal desmoplasia, polyphenotypic differentiation and EWSR1-WT1 gene fusion”, unusual morphological features, such as spindle, rhabdoid and epithelioid cell morphology and the presence of a glandular epithelial component, have been rarely described [21]. The present case contributes to widen the morphological spectrum of DSRCT. To the best of our knowledge, only a few cases of DSRCT with unusual morphology are reported in the literature [21,22,23]. Ordonez in 1998 [21] reported a series of 39 cases of DSRCTs, 6 of which showed extensive or focal spindle cell morphology, while the absence of stromal desmoplasia was found only in 1 case; this latter tumor showed an insular growth pattern with thin fibrovascular septa, mimicking a well-differentiated neuroendocrine tumor. In addition, Alaggio et al. [22] described two peculiar cases of DSRCT in an 11-year-old and in a 9-year-old boy, morphologically resembling to leiomyosarcomas; interestingly, these tumors [22] showed strong immunoreactivity for desmin and keratins, as expected for DSRCT, but also smooth muscle markers, unusual for this entity. Molecular analyses for the fusion transcript *t(11;22)(p13;q12)* showed in both tumors the presence of the chimeric transcript EWS-WT1 [21], a characteristic finding of DSRCT. Notably, both patients had a significantly better prognosis than expected for a classic DSRCT, raising the question of whether the presence of the EWS-WT1 translocation is per se sufficient to render a diagnosis of DSRCT even in the absence of the typical clinico-pathological features.

Apart from the fibrosarcomatous-like morphology and the absence of desmoplastic stroma, the clinical presentation and the polyphenotypic immunoprofile in the present case were strongly suggestive of DSRCT. Molecular studies were crucial in confirming the diagnosis. Notably, the patient died 9 months after the diagnosis, suggesting that also the outcome was consistent with DSRCT. An intriguing finding of our case was that chimeric transcripts found by RT-PCR analysis were composed of an unusual in-frame junction of exon 9 of *EWS* to exon 7 of *WT1*. It is well accepted that, although the *EWS-WT1* fusion transcript of DSRCT usually consists of the first 7 exons of the *EWS* gene fused to the last 3 exons the *WT1* gene (*EWS-WT1* 7/8) [24], multiple non-conventional breakpoints for the *t(11;22)(p13;q12)* translocation have been reported [25,26,27,28,29], consisting of additional exons from *EWS* gene with conservation of the *WT1* complement (*EWS-WT1* 8/8, 9/8, and 10/8). It still remains unknown if the variant of *EWS-WT1* (9/7) transcript (exons 1 to 9 of *EWS* fused to exons 7 to 9 of *WT1*) found in the present case, may be related to the unusual morphology of the tumor. Murphy et al. [28] described a soft tissue DSRCT, containing 2 fusion transcripts, deleted for *WT1* exons 9 and 10. In particular, the first fusion transcript contained the exon 7 of *EWS* fused to exon 8 of *WT1*; the exon 7 and part of exon 8 of *EWS* translocated to exon 3 of *WT1* were present in the second variant. These authors [28] also compared the abovementioned molecular results to those from 5 control cases of intra-abdominal DSRCTs, 1 of which presented a variant *EWS-WT1* 9/8 transcript, containing exons 1 to 9 of *EWS* and exons 8 to 10 of *WT1*, similar to that found in our case and previously reported by Chan et al. [32].

## 5. Conclusions

In conclusion, we presented a case of DSRCT with conventional clinical presentation and outcome, but with unusual morphology (pure spindle cell morphology; absence of desmoplastic stroma) and molecular findings (variant of *EWS-WT1* fusion transcript). Further studies are required to better establish whether the presence of a novel fusion transcript could, at least partially, explains the unusual morphology of our unique case.

## References

[1] Gerald W.L., Rosai J. (1989). Case 2. Desmoplastic small cell tumor with divergent differentiation. Pediatr. Pathol..

[2] Gerald W.L., Ladanyi M., de Alava E., Cuatrecasas M., Kushner B.H., LaQuaglia M.P., Rosai J. (1998). Clinical, pathologic, and molecular spectrum of tumors associated with t(11;22)(p13;q12): Desmoplastic small round-cell tumor and its variants. J. Clin. Oncol..

[3] Sawyer J.R., Tryka A.F., Lewis J.M. (1992). A novel reciprocal chromosome translocation t(11;22)(p13;q12) in an intraabdominal desmoplastic small round-cell tumor. Am. J. Surg. Pathol..

[4] Mora J., Modak S., Cheung N.K., Meyers P., de Alava E., Kushner B., Magnan H., Tirado O.M., Laquaglia M., Ladanyi M. (2015). Desmoplastic small round cell tumor 20 years after its discovery. Future Oncol..

[5] Gerald W.L., Miller H.K., Battifora H., Miettinen M., Silva E.G., Rosai J. (1991). Intra-abdominal desmoplastic small round-cell tumor. Report of 19 cases of a distinctive type of high-grade polyphenotypic malignancy affecting young individuals. Am. J. Surg. Pathol..

[6] Thway K., Noujaim J., Zaidi S., Miah A.B., Benson C., Messiou C., Jones R.L., Fisher C. (2016). Desmoplastic Small Round Cell Tumor: Pathology, Genetics, and Potential Therapeutic Strategies. Int. J. Surg. Pathol..

[7] Brodie S.G., Stocker S.J., Wardlaw J.C., Duncan M.H., McConnell T.S., Feddersen R.M., Williams T.M. (1995). EWS and WT-1 gene fusion in desmoplastic small round cell tumor of the abdomen. Hum. Pathol..

[8] Rekhi B., Ahmed S., Basak R., Qureshi S.S., Desai S.S., Ramadwar M., Desai S.B., Kurkure P., Jambhekar N.A. (2012). Desmoplastic small round cell tumor-clinicopathological spectrum, including unusual features and immunohistochemical analysis of 45 tumors diagnosed at a tertiary cancer referral centre, with molecular results t(11;22)(p13;q12) (EWS-WT1) in select cases. Pathol. Oncol. Res..

[9] Zhang J., Xu H., Ren F., Yang Y., Chen B., Zhang F. (2014). Analysis of clinicopathological features and prognostic factors of desmoplastic small round cell tumor. Pathol. Oncol. Res..

[10] Morani A.C., Bathala T.K., Surabhi V.R., Yedururi S., Jensen C.T., Huh W.W., Prasad S., Hayes-Jordan A. (2019). Desmoplastic Small Round Cell Tumor: Imaging Pattern of Disease at Presentation. AJR Am. J. Roentgenol..

[11] Ertoy Baydar D., Armutlu A., Aydin O., Dagdemir A., Yakupoglu Y.K. (2020). Desmoplastic small round cell tumor of the kidney: A case report. Diagn. Pathol..

[12] Hatanaka K.C., Takakuwa E., Hatanaka Y., Suzuki A., IIzuka S., Tsushima N., Mitsuhashi T., Sugita S., Homma A., Morinaga S. (2019). Desmoplastic small round cell tumor of the parotid gland-report of a rare case and a review of the literature. Diagn. Pathol..

[13] He X.R., Liu Z., Wei J., Li W.J., Liu T. (2019). Primary desmoplastic small round cell tumor in the left orbit: A case report and literature review. Int. Ophthalmol..

[14] Lal D.R., Su W.T., Wolden S.L., Loh K.C., Modak S., La Quaglia M.P. (2005). Results of multimodal treatment for desmoplastic small round cell tumors. J. Pediatr. Surg..

[15] Al-Ibraheemi A., Broehm C., Tanas M.R., Horvai A.E., Rubin B.P., Cheah A.L., Thway K., Fisher C., Bahrami A., Folpe A.L. (2019). Desmoplastic Small Round Cell Tumors With Atypical Presentations: A Report of 34 Cases. Int. J. Surg. Pathol..

[16] Magro G., Longo F.R., Angelico G., Spadola S., Amore F.F., Salvatorelli L. (2015). Immunohistochemistry as potential diagnostic pitfall in the most common solid tumors of children and adolescents. Acta Histochem..

[17] Parenti R., Salvatorelli L., Musumeci G., Parenti C., Giorlandino A., Motta F., Magro G. (2015). Wilms’ tumor 1 (WT1) protein expression in human developing tissues. Acta Histochem..

[18] Salvatorelli L., Parenti R., Leone G., Musumeci G., Vasquez E., Magro G. (2015). Wilms tumor 1 (WT1) protein: Diagnostic utility in pediatric tumors. Acta Histochem..

[19] Salvatorelli L., Calabrese G., Parenti R., Vecchio G.M., Puzzo L., Caltabiano R., Musumeci G., Magro G. (2020). Immunohistochemical Expression of Wilms’ Tumor 1 Protein in Human Tissues: From Ontogenesis to Neoplastic Tissues. Appl. Sci..

[20] Piombino E., Broggi G., Barbareschi M., Castorina S., Parenti R., Bartoloni G., Salvatorelli L., Magro G. (2021). Wilms’ Tumor 1 (WT1): A Novel Immunomarker of Dermatofibrosarcoma Protuberans-An Immunohistochemical Study on a Series of 114 Cases of Bland-Looking Mesenchymal Spindle Cell Lesions of the Dermis/Subcutaneous Tissues. Cancers.

[21] Ordóñez N.G. (1998). Desmoplastic small round cell tumor: I: A histopathologic study of 39 cases with emphasis on unusual histological patterns. Am. J. Surg. Pathol..

[22] Alaggio R., Rosolen A., Sartori F., Leszl A., d’Amore E.S., Bisogno G., Carli M., Cecchetto G., Coffin C.M., Ninfo V. (2007). Spindle cell tumor with EWS-WT1 transcript and a favorable clinical course: A variant of DSCT, a variant of leiomyosarcoma, or a new entity? Report of 2 pediatric cases. Am. J. Surg. Pathol..

[23] Lae M.E., Roche P.C., Jin L., Lloyd R.V., Nascimento A.G. (2002). Desmoplastic small round cell tumor: A clinicopathologic, immunohistochemical, and molecular study of 32 tumors. Am. J. Surg. Pathol..

[24] Gerald W.L., Rosai J., Ladanyi M. (1995). Characterization of the genomic breakpoint and chimeric transcripts in the EWS-WT1 gene fusion of desmoplastic small round cell tumor. Proc. Natl. Acad. Sci. USA.

[25] Werner H., Idelman G., Rubinstein M., Pattee P., Nagalla S.R., Roberts C.T. (2007). A novel EWS-WT1 gene fusion product in desmoplastic small round cell tumor is a potent transactivator of the insulin-like growth factor-I receptor (IGF-IR) gene. Cancer Lett..

[26] Nakanishi Y., Oinuma T., Sano M., Fuchinoue F., Komatsu K., Seki T., Obana Y., Tabata M., Kikuchi K., Shimamura M. (2006). Coexpression of an unusual form of the EWS-WT1 fusion transcript and interleukin 2/15 receptor betamRNA in a desmoplastic small round cell tumour. J. Clin. Pathol..

[27] Hamazaki M., Okita H., Hata J., Shimizu S., Kobayashi H., Aoki K., Nara T. (2006). Desmoplastic small cell tumor of soft tissue: Molecular variant of EWS-WT1 chimeric fusion. Pathol. Int..

[28] Murphy A.J., Bishop K., Pereira C., Chilton-MacNeill S., Ho M., Zielenska M., Thorner. P.S. (2008). A new molecular variant of desmoplastic small round cell tumor: Significance of WT1 immunostaining in this entity. Hum. Pathol..

[29] Shimizu Y., Mitsui T., Kawakami T., Ikegami T., Kanazawa C., Katsuura M., Obata K., Yamagiwa I., Hayasaka K. (1998). Novel breakpoints of the EWS gene and the WT1 gene in a desmoplastic small round cell tumor. Cancer Genet. Cytogenet..

[30] Rieux-Laucat F. (2017). What’s up in the ALPS. Curr. Opin. Immunol..

[31] Lee Y.S., Hsiao C.H. (2007). Desmoplastic small round cell tumor: A clinicopathologic, immunohistochemical and molecular study of four patients. J. Formos. Med. Assoc..

[32] Chan A.S., MacNeill S., Thorner P., Squire J., Zielenska M. (1999). Variant EWS-WT1 chimeric product in the desmoplastic small round cell tumor. Pediatr. Dev. Pathol..

